# Authors’ Response to Peer Reviews of “Levels and Predictors of Knowledge, Attitudes, and Practices Regarding Contraception Among Female TV Studies Undergraduates in Nigeria: Cross-Sectional Study”

**DOI:** 10.2196/72947

**Published:** 2025-05-08

**Authors:** Hadizah Abigail Agbo, Philip Adewale Adeoye, Danjuma Ropzak Yilzung, Jawa Samson Mangut, Paul Friday Ogbada

**Affiliations:** 1 Department of Community Medicine, Jos University Teaching Hospital, Lamingo, Jos, Plateau State, Nigeria, +234 8062081617; 2 Department of Community Medicine, University of Jos, Jos, Plateau State, Nigeria; 3 College of Health Sciences, University of Jos, Jos, Plateau State, Nigeria

**Keywords:** knowledge, attitudes, practice, contraception, regression, cross-sectional, females, students, Nigeria

*This is the authors’ response to peer-review reports of “Levels and Predictors of Knowledge, Attitudes, and Practices Regarding Contraception Among Female TV Studies Undergraduates in Nigeria: Cross-Sectional Study.”*

## Round 1 Review

### Reviewer Q [1]

#### General Comments

*Dear Authors,*

*Thank you very much for undertaking the study [2] titled “Levels and predictors of knowledge, attitude and practice of contraception among female TV undergraduates in Nigeria: a cross-sectional study” and submitting the manuscript to JMIR. The study findings are important for family planning program implementation targeting young students. I have the following comments and observations for improving your manuscript for consideration of publishing.*

**Response:** We would like to thank the reviewer for the kind words and helpful comments. We are indeed grateful.

#### Specific Comments

##### Major Comments

*Introduction: line 50: “youth”: Indicate age group.*

**Response:** Done. The age range (17‐35 years) of the study participants falls within the definition of youth by the National Baseline Youth Survey of Nigeria and the African Youth Charter [3,4]. The classification used for teenagers/adolescents agrees with the World Health Organization definition and those used in the literature on adolescents [5,6]. The classification of young adults used agrees with that of Statistics Canada [7].

*Line 52: “Utilization is higher”: Not clear what the utilization was for.*

**Response:** Revised to “contraceptive utilization rate.”

*Study population: limitation: gender biased. Male involvement and attitude are equally important regarding sexually transmitted infections, particularly for male methods like use of condoms. This needs to be mentioned as a limitation of the study.*

**Response:** Done.

*Tables all: Hastily, one sentence is used for describing findings in a table. Need to elaborate more. Further comments below.*

**Response:** Tables are now more elaborate in narration.

*Table 1: Rephrase the “Marital status” indicator; the data does not give the status of marriage!*

**Response:** The original marital status categorization on the data collection form includes single, married, separated, divorced, and widowed. However, after the data collection, only single (n=197, 96.8%), married (n=19, 8.8%), and separated (n=1, 0.5%) were reported. Since only 1 study participant reported herself as separated and this group is similar to singles by not living with their spouse, they were, therefore, merged to ease the interpretation of data and to reflect the impact of living with a spouse on contraceptive attitude and use. Therefore, I have rephrased the marital status grouping as married and single/separated.

*Table 2: Indicate what is meant by poor, good, etc knowledge/attitude; cite measurement scale here.*

**Response:** It has already been stated in the Data Management and Analysis subsection of the Methodology section. The classification is based on the use of the average scores. This is dependent on whether they are normally distributed or not: when they are normally distributed, mean (SD) was used, but when not normally distributed, median (IQR) was reported. Good knowledge, attitude, and practice are at least the average scores; while poor knowledge, attitude, and practice are less than the average scores. This approach to categorization is important to prevent the “ceiling effect” in subjective socially biased items in surveys. Therefore, the categorization scale has been indicated within the narration of the result as requested.

*Table 3: Need to mention if this was an open-ended or structured question.*

**Response:** It has already been stated under the data collection methods: “Data was collected from female students of NTA TV College Jos by the research team using a semi-structured self-administered questionnaire...” The questionnaire is semistructured and contains both structured and open-ended items.

*Table 4: Cite the indicators used for measuring attitude toward use of contraception.*

**Response:** Indicators include those items explored in the secondary analysis of this data, which has been posted on a preprint server to provide insight into the items driving the reported levels and predictors of contraception reported in this study [8].

*Table 5: The predictor of not engaging in sex may be reflected well in statistical analysis, but what is the significance in real life? Why would those who had never engaged in sex have used contraception?*

**Response:** Though it might not be relatively acceptable and valid to ask those who have never had sexual intercourse about contraceptive use, the researchers were prompted to generally ask this question due to the prevalence of intimate partner violence among unmarried and separated people with the prevalence of sexual violence being one of the highest in the country; much earlier first sexual experiences among the age range in the study population in the study area, region, and country, with first sexual experience not forced; increasing use of contraception for other purposes other than family planning among the study population; increasing liberal attitudes toward contraceptive use; and the social desirability bias that can be produced with questions surrounding sex and contraception [9-11]. We were justified by the time we explored the result of the responses and the inconsistencies reported by the study participants; some of the results were added to a preprint published earlier this year, but 73.7% of this study population reported having had sex, and a higher proportion (94.9%) of the total study population reported history of unplanned pregnancy [8,12]. That might have been the reason, among many others, why many other published studies have included the same item in questionnaires for all study participants irrespective of declared sexual activity status [13-15].

*Discussion: Mention the rate of use of emergency contraceptive pills (ECPs) also. This is increasing in many societies. Policy makers/planners are often not aware of the need for ECPs to include a supply of ECPs in a program.*

**Response:** The report to reflect the use of emergency contraception has been expanded in the Result section. Due to the small proportion of study participants using emergency contraception, they have been merged with those reporting implants and many unnamed forms of contraception in the “others category.” Further discussion on emergency contraception use has been included in the Discussion also.

*A recommendation like “There may be a need to use social marketing 42 approaches to make these contraceptives available to young people to bypass the stigma they experienced while accessing 43 contraceptives from traditional sources of contraceptives” is not supported by any finding or data of the study. Rather this raises a question of bias on jumping to a solution through a particular channel. Let the program planners find out the way to resolve the issue of information availability.*

**Response:** The discussion on social marketing implications under contraceptive use has been removed following the recommendation of the reviewer. However, social marketing is a veritable tool in ensuring improved access to contraception for young people using marketing approaches. Its recognized ability to increase use also prompted its inclusion in the Nigeria Demographic and Health Survey 2018 for the first time, where women of reproductive age (15‐49 years) in the country (including the study area) were asked about the use of social marketing to access contraception [9].

*Highlights: Move the highlights to the Discussion section because this is a summary of the findings.*

**Response:** Done.

*Conclusion: Rewrite the conclusion, elaborating on recommendations per results of the study.*

**Response:** Done. Other important recommendations have been added to the discussion of important results. Discussions usually include a statement on a result, comparison/variation with prior studies, and reasons for the similarities/variations and implications for policy and practice.

### Reviewer BO [16]

#### Specific Comments

##### Major Comments

*1. The sampling technique used in this paper should be more detailed than it is. Respondents were said to have been selected by balloting from the 6 levels. Was it equal allocation per level, or was it proportionate allocation considering that it is not likely that there were the same number of students in each level?*

**Response:** A detailed sampling has been reported in the work as requested by the reviewer.

*2. State the age ranges of a teenager and that of a young adult in your methodology that informed the categorization in the Results.*

**Response:** Done.

*3. Living with a spouse and not living with a spouse was considered for marital status in your study as opposed to being single, married, etc. Clarify why this is so.*

**Response:** The original marital status categorization on the data collection form included single, married, separated, divorced, and widowed. However, after the data collection, only single (n=197, 96.8%), married (n=19, 8.8%), and separated (n=1, 0.5%) were reported. Since only 1 study participant reported herself as separated and this group is similar to singles by not living with their spouse, they were merged to ease the interpretation of data and to reflect the impact of living with a spouse on contraceptive attitude and use. Therefore, I have rephrased the marital status grouping as married and single/separated.

*4. The public health implications of some of the findings were omitted in the Discussion. This should be included. Its importance cannot be overemphasized.*

**Response:** Done.

##### Minor Comments

*5. Abstract: The last sentence in the Methods is hanging. Kindly complete it.*

**Response:** Done.

*6. Grammatical issues: Tenses: Future and present tenses were used where past tense should have been used in the methodology (lines 12 and 28). Present tense was used in multiple places in the Discussion where past tense should have been used.*

**Response:** Done.

*7. Reference list: In the Vancouver referencing style, the month of publication should not appear as it did in some references like 7, 11, and 12. The date accessed/cited was written in some and not in others like 9, 10, 13, and 16. Really old references like reference 24, which is 14 years old, should be replaced by more current ones.*

**Response:** Done. “Month of publication” as seen in some journal references had been removed from the reference list.

Revised to conform to stated format. Months of access were included in websites as seen in references 16, 17, 18, 21, 22, 35, and 36; while they were not included in references 1, 4, 6, 9, 10, 13, 45, and 48 because access dates are not necessarily included in reports. Also, to prevent unnecessary errors in referencing, Mendeley referencing software was used.

Really old references (2) have been replaced [17,18]. Others (3) were left because they are either a charter or government document that contribute to a definition [3,4] or milestone document [19].

## Abbreviations

ECP: emergency contraceptive pill
